# sCD40 and sCD40L as candidate biomarkers of rheumatic diseases: a systematic review and meta-analysis with meta-regression

**DOI:** 10.3389/fimmu.2025.1479904

**Published:** 2025-03-19

**Authors:** Angelo Zinellu, Arduino A. Mangoni

**Affiliations:** ^1^ Department of Biomedical Sciences, University of Sassari, Sassari, Italy; ^2^ Discipline of Clinical Pharmacology, College of Medicine and Public Health, Flinders University, Adelaide, SA, Australia; ^3^ Department of Clinical Pharmacology, Flinders Medical Centre, Southern Adelaide Local Health Network, Adelaide, SA, Australia

**Keywords:** sCD40, sCD40L, B cells, T cells, rheumatic diseases, inflammation, autoimmunity, disease activity

## Abstract

There is an ongoing search for novel biomarkers to enhance diagnosing and monitoring patients with rheumatic diseases (RDs). We conducted a systematic review and meta-analysis to investigate the potential role of the soluble cluster of differentiation 40 (sCD40) and sCD40 ligand (sCD40L), involved in humoral and cellular immune response, as candidate biomarkers of RDs. We searched PubMed, Web of Science, and Scopus from inception to 30 June 2024 for studies investigating circulating sCD40 and sCD40L concentrations in RD patients and healthy controls. We assessed the risk of bias using the Joanna Briggs Institute Critical Appraisal Checklist for analytical studies and the certainty of evidence using the Grades of Recommendation, Assessment, Development and Evaluation Working Group system. Compared to controls, RD patients had significantly higher sCD40L (31 studies; standard mean difference, SMD=0.87, 95% CI 0.60 to 1.13, p<0.001; low certainty of evidence) and sCD40 (five studies; SMD=1.32, 95% CI 0.45 to 2.18, p=0.003; very low certainty of evidence) concentrations. In meta-regression and subgroup analysis, the effect size of the between-group differences in sCD40L was significantly associated with sample size, mean RD duration, specific RD, biological matrix assessed, and analytical method used. By contrast, there were no associations with age, sex, C-reactive protein, erythrocyte sedimentation rate, use of disease-modifying antirheumatic drugs or glucocorticoids, or geographical location. There were no significant differences in sCD40L concentrations between RD patients with and without active disease (eight studies; SMD=0.12, 95% CI -0.09 to 0.33, p=0.26; very low certainty). By contrast, sCD40 concentrations were significantly higher in RD patients with active disease (three studies; SMD=0.36, 95% CI 0.08 to 0.84, p=0.013; very low certainty). Our systematic review and meta-analysis suggests the potential role of sCD40 and sCD40L as candidate biomarkers to detect the presence of RDs (sCD40 and sCD40L) and monitor disease activity (sCD40). Large, appropriately designed prospective studies in a wide range of RDs are warranted to investigate whether measuring sCD40 and sCD40L can significantly improve the performance of currently available diagnostic criteria and serological biomarkers. (PROSPERO registration number: CRD42024577430).

**Systematic review registration:**

https://www.crd.york.ac.uk/PROSPERO/view/CRD42024577430, identifier PROSPERO CRD42024577430.

## Introduction

Early diagnosis and treatment significantly improve the quality of life and prognosis in patients with rheumatic diseases (RDs), a group of autoimmune (e.g., rheumatoid arthritis, RA), autoimmune-autoinflammatory (e.g., Behcet’s disease, BD), or autoinflammatory (e.g., familial Mediterranean fever, FMF) conditions affecting various organs and systems (1–9). However, diagnosing early, subtle forms of RDs remains challenging, particularly for nonspecialists. This vexing issue has stimulated research to identify novel biomarkers of disease to aid clinical evaluation and management (10–15). Ideally, such biomarkers should adequately reflect alterations of critical pathways regulating immune response and inflammation (16–18).

The cluster of differentiation 40 (CD40)/CD40 ligand (CD40L) dyad is a pivotal regulator of the humoral and cellular immune response (19, 20). CD40 is a membrane glycoprotein that is part of the tumor necrosis factor (TNF) receptor superfamily (21). CD40 is expressed in many cells, including B cells, endothelial cells, epithelial cells, monocytes, macrophages, fibroblasts, and dendritic cells (19–21). CD40L, also a glycoprotein and member of the TNF superfamily, is transiently expressed in activated T cells, mainly the CD4^+^ T-cell subset, basophils, mast cells, eosinophils, natural killer cells, and platelets (22). The CD40L-mediated activation of CD40 favors the growth and differentiation of B cells, immunoglobulin class switching, and antigen-presenting cell activation by inducing cytokine synthesis (19, 20). The CD40L-mediated activation of CD40 also induces short-term activation and cytokine production in T cells. Following cell activation, CD40L translocates to the cell surface as membrane CD40L (mCD40L). CD40L also exists as a soluble form (sCD40L) that is generated either from enzymatic cleavage of mCD40L or intracellular CD40L. Both mCD40L and sCD40L are biologically active (19, 20). Two forms of CD40 also exist, membrane (mCD40) and soluble (sCD40). sCD40 is formed by alternative splicing in the cytoplasm or following proteolysis of mCD40 following ligation with CD40L. Notably, sCD40 antagonizes the effects of CD40 (19, 20). Therefore, measuring circulating sCD40 and sCD40L may be helpful in characterizing the immune response in different types of RDs, complementing the information provided by clinical assessment and available diagnostic criteria and serological biomarkers.

Therefore, we investigated the potential role of sCD40 and sCD40L as candidate biomarkers by conducting a systematic review and meta-analysis of studies reporting their concentrations in serum or plasma in RD patients and healthy controls. We further investigated possible associations between the effect size of the between-group differences and various study and patient variables, including demographic characteristics, type of RD, mean RD duration, conventional inflammatory markers (i.e., C-reactive protein (CRP) and erythrocyte sedimentation rate (ESR), and use of disease-modifying antirheumatic drugs (DMARDs) and corticosteroids.

## Materials and methods

### Search strategy, screening, and study selection

We systematically searched PubMed, Web of Science, and Scopus, from inception to 30 June 2024, for relevant articles using the following terms (please refer to **Supplementary Table 1** for additional details regarding the search strategy): “soluble cluster of differentiation 40” OR “sCD40” OR “soluble CD40” OR “sCD40L” OR “soluble CD40L” OR “sCD40 ligand” OR “sCD154” AND “rheumatic diseases” OR “rheumatoid arthritis” OR “psoriatic arthritis” OR “ reactive arthritis” OR “ankylosing spondylitis” OR “systemic lupus erythematosus” OR “systemic sclerosis” OR “scleroderma” OR “Sjogren’s syndrome” OR “connective tissue diseases” OR “vasculitis” OR “Behçet’s disease” OR “idiopathic inflammatory myositis” OR “polymyositis” OR “dermatomyositis” OR “gout” OR “pseudogout” OR “ systemic vasculitis” OR “ANCA-associated vasculitis” OR “Takayasu arteritis” OR “polyarteritis nodosa” OR “osteoarthritis” OR “fibromyalgia” OR “granulomatous polyangiitis” OR “Henoch-Schonlein purpura” OR “Wegener’s granulomatosis” OR “familial Mediterranean fever” OR “polymyalgia rheumatica”.

Initially, two investigators independently screened each abstract for relevance. Then, they independently reviewed the full text of each article. The inclusion criteria were: (i) the measurement of circulating sCD40L and/or sCD40 concentrations, (ii) the comparison between RD patients and healthy controls and/or between RD patients with and without active disease (case-control design), (iii) the inclusion of participants aged ≥18 years, (iv) the use of English language, (v) the recruitment of at least ten RD patients and/or controls, and (vi) the availability of the full text of the publication. The exclusion criteria were: (i) *in vitro* or animal studies, (ii) the inclusion of participants under 18 years, and (iii) the inclusion of less than ten RD patients and/or controls. The references of the retrieved articles were hand-searched to identify additional studies.

The two investigators independently extracted the following information into separate electronic sheets for further analysis: first author, year of publication, country where the study was conducted, RD type, mean RD duration, number of participants, age, male-to-female ratio, CRP, ESR, use of DMARDs or glucocorticoids, sample matrix assessed (serum or plasma), and analytical method used. Any disagreement was resolved by a third investigator.

We assessed the risk of bias of each article using the Joanna Briggs Institute Critical Appraisal Checklist for analytical studies (23) and the level of the certainty of evidence using the Grades of Recommendation, Assessment, Development and Evaluation (GRADE) Working Group system (24). We wholly adhered to the Preferred Reporting Items for Systematic reviews and Meta-Analyses (PRISMA) 2020 statement (**Supplementary Table 1**) (25). We registered the study protocol in the International Prospective Register of Systematic Reviews (PROSPERO registration number: CRD42024577430).

### Statistical analysis

We generated forest plots of standardized mean differences (SMDs) and 95% confidence intervals of sCD40L and sCD40 concentrations between RD patients and healthy controls and between RD patients with and without active disease (a p-value <0.05 was considered statistically significant). Medians and interquartile ranges were extracted from graphs using the Graph Data Extractor software (San Diego, CA, USA). Using published methods, we extrapolated the means and standard deviations from the medians and interquartile or full ranges (26). We used the Q statistic (a p-value <0.10 was considered statistically significant) to assess the heterogeneity of SMD across studies. A low, moderate, and high heterogeneity was indicated by I^2^ values of ≤ 25%, >25% and <75%, and ≥75%, respectively (27, 28). We used a random-effect model based on the inverse-variance method in the presence of high heterogeneity (29). We conducted sensitivity analyses to test the stability of the meta-analysis results and assessed publication bias using standard methods (a p-value <0.05 was considered statistically significant) (30–33). We conducted meta-regression and subgroup analyses to investigate associations between the effect size and year of publication, country where the study was conducted, RD type, mean RD duration, sample size, age, male-to-female ratio, CRP, ESR, use of DMARDs and/or glucocorticoids, sample matrix assessed, and analytical method used. All statistical analyses were performed using Stata 14 (Stata Corp., College Station, TX, USA).

## Results

**Figure 1:**
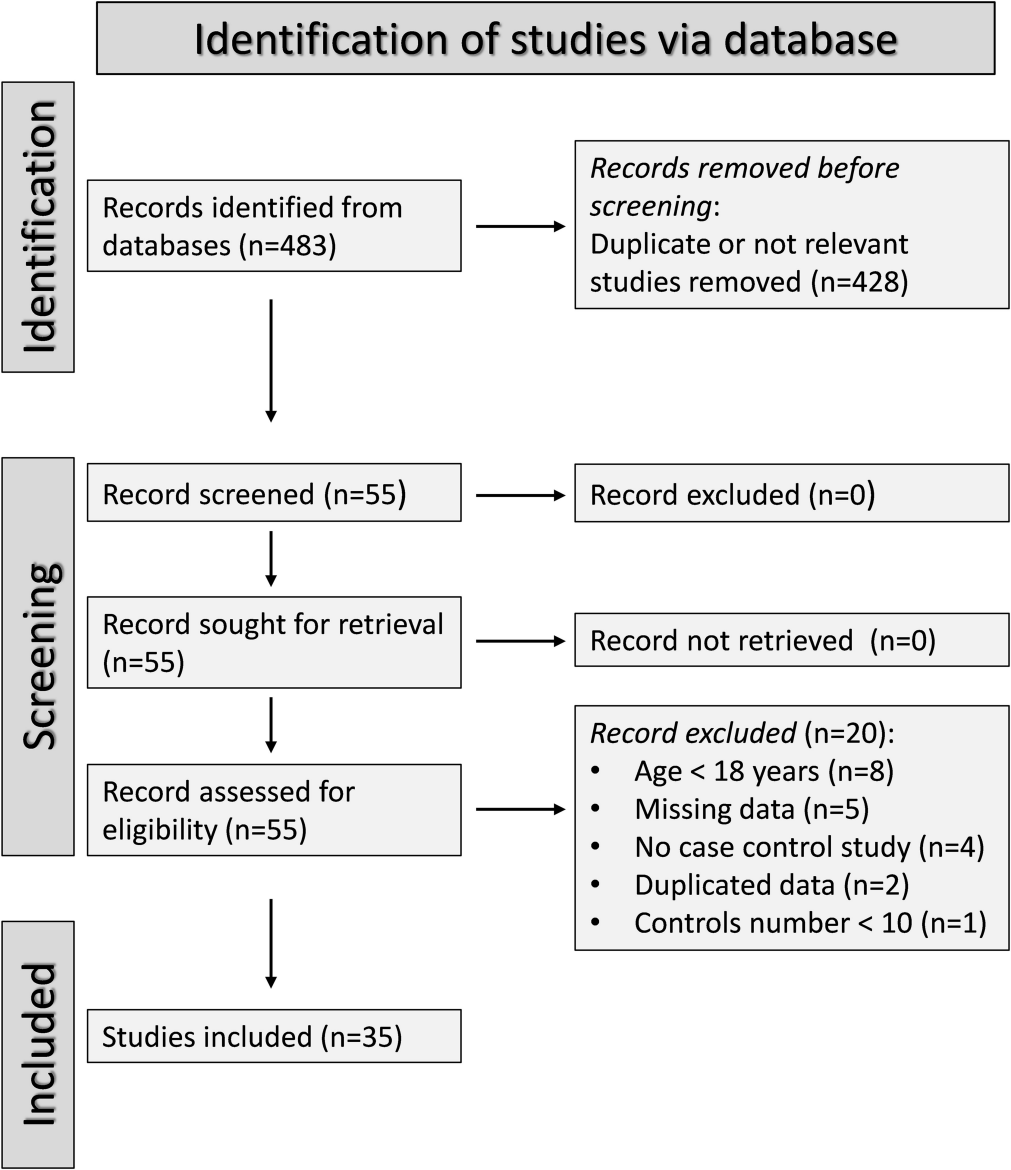
PRISMA 2020 flow diagram of screening and study selection.

**Figure 1** describes the flow chart of the screening process and study selection. We initially identified 483 articles. After the first screening, we excluded 428 articles because they reported duplicate or irrelevant information. After full-text revision of the remaining 55 articles, we excluded eight studies because they enrolled participants under 18 years, five because of missing data, four because of a different study design, two because of duplicate data, and one because the number of controls was less than ten. Thus, we selected 35 studies for analysis (34–68). Their characteristics are described in **Table 1**. Given the cross-sectional design of the studies identified in our search, we ranked the initial level of the certainty of evidence as low (level 2).

**Table 1:** Characteristics of studies investigating sCD40L and sCD40 in patients with rheumatic diseases and healthy controls.

Study	Controls				Patients with rheumatic diseases				Disease type	MDD (Years)
	n	Age (Years)	M/F	sCD40L (Mean ± SD)	n	Age (Years)	M/F	sCD40L (Mean ± SD)		
Kato K et al., 1999, Japan (34)	21	NR	NR	290 ± 340	26	NR	NR	6800 ± 4300	SLE	NR
Vakkalanka RK et al., 1999, USA (35)	23	NR	NR	0.025 ± 0.04	66	NR	NR	2.61 ± 2.15	SLE	NR
Tamura N et al., 2001, Japan (36)	20	47.8	6/14	0.17 ± 0.19	39	52.5	10/29	2.44 ± 3.18	RA	NR
Allanore Y et al., 2005, France (37)	20	49.6	2/18	81.5 ± 18.2	50	57	6/44	2180 ± 1718	SSc	7
Goules A et al. (a) 2006, Greece (38)	17	NR	NR	38 ± 20	23	NR	NR	57.6 ± 151	SLE	NR
Goules A et al. (b) 2006, Greece (38)	17	NR	NR	38 ± 20	23	NR	NR	61.4 ± 131	pSS	NR
Goules A et al. (c) 2006, Greece (38)	17	NR	NR	38 ± 20	16	NR	NR	52.6 ± 52	RA	NR
Von Feldt JM et al., 2006, USA (39)	142	43.6	0/142	9.7 ± 4.4	152	43.3	0/152	7.2 ± 4.4	SLE	11.1
Ciferská H et al., 2007, Czech Republic (40)	15	matched	matched	2.96 ± 1.39	65	37	3/65	7.4 ± 6.7	SLE	NR
Nomura K et al., 2008, Japan (41)	30	43	11/19	3.9 ± 2.2	42	48.4	7/35	6.3 ± 3.1	SSc	NR
Pamuk GE et al., 2008, Turkey (42)	19	49.1	6/13	2.98 ± 1.2	27	51.6	6/21	5.29 ± 2.1	RA	7.35
Cella G et al., 2009, Italy (43)	18	matched	matched	245.74 ± 111.8	18	53.8	5/13	1685.33 ± 866	CTD	11.8
De Sanctis JB et al., 2009, Venezuela (44)	100	33	15/85	3.9 ± 1.2	60	32.1	5/55	8.6 ± 2.8	SLE	NR
ElGendi SS et al., 2009, Egypt (45)	20	NR	NR	1.3 ± 0.61	47	25.26	NR	4.97 ± 4.35	SLE	2.57
Pamuk GE et al., 2009, Turkey (46)	20	45.7	6/14	0.9 ± 0.6	20	46.3	8/12	1.12 ± 0.6	PsA	NR
Sellam J eta l. (a) 2009, France (47)	44	41.5	7/37	133.6 ± 25.6	43	55.75	1/42	233 ± 182.1	pSS	10.25
Sellam J eta l. (b) 2009, France (47)	44	41.5	7/37	133.6 ± 25.6	20	43.5	1/19	262.6 ± 263.5	SLE	10.88
Sellam J eta l. (c) 2009, France (47)	44	41.5	7/37	133.6 ± 25.6	26	53.5	5/21	345.7 ± 336.5	RA	13.12
Sari I et al., 2010, Turkey (48)	38	36.4	11/27	8.56 ± 5.33	44	38.8	10/34	8.73 ± 3.73	AS	NR
Fernández Bello I et al., 2012, Spain (49)	28	40	8/20	179 ± 294	30	42	8/22	2228 ± 1485	BD	14
Orum H et al., 2012, Turkey (50)	22	33.1	8/14	1.1 ± 0.4	59	36.7	11/48	1.5 ± 0.7	AS	NR
Pamuk GE et al. (a) 2014, Turkey (51)	94	40.1	16/78	2.27 ± 1.2	100	38.9	19/81	2.79 ± 1.7	RA	5.1
Pamuk GE et al. (b) 2014, Turkey (51)	94	40.1	16/78	2.27 ± 1.2	81	55.2	7/74	2.08 ± 2.08	SLE	9.15
Cantarini L et al., 2016, Italy (53)	35	NR	NR	2016.16 ± 888.35	27	45.7	12/15	3445.78 ± 967.09	BD	13.59
Yalçınkaya Y et al., 2016, Turkey (54)	20	NR	NR	24620 ± 13051	72	44.9	6/66	27847 ± 33315	SSc	NR
Kim KJ et al., 2017, South Korea (55)	37	NR	NR	41 ± 59	241	34.8	19/222	53.3 ± 22.4	SLE	6.25
Perazzio SF et al., 2017, Brazil (56)	30	35.6	12/18	6717 ± 6545	61	29.4	32/29	14119 ± 4442	BD	10
Petrackova A et al., 2017, Czech Republic (57)	23	40	8/15	527 ± 61	75	43.3	9/66	735 ± 142	SLE	15.25
Stanek A et al., 2017, Poland (58)	48	46.63	0/48	5.54 ± 2.37	48	46.06	0/48	8.93 ± 3.74	AS	NR
Willis R et al. (a) 2017, USA (59)	30	43.5	5/25	17.9 ± 11.4	45	44	1/44	343 ± 382	SLE	6.8
Willis R et al. (b) 2017, USA (59)	30	43.5	5/25	17.9 ± 11.4	267	47.6	15/252	2839 ± 4385	SLE	16.9
Román Fernández IV et al., 2019, Mexico (61)	10	matched	0/10	54.34 ± 7.4	38	48.2	0/38	89.01 ± 44.6	RA	8.35
Sodergren A et al., 2019, Sweden (62)	40	48.1	8/32	23.6 ± 6.5	71	51.5	10/61	21.9 ± 7.3	RA	1.34
Zamora C et al., 2019, Spain (64)	16	49.06	4/12	50.58 ± 25.79	21	50.67	0/21	691.3 ± 268.7	SLE	12.41
Venerito V et al., 2020, Italy (65)	20	50	4/16	2575 ± 843.1	27	58.4	11/16	5364 ± 2025	PsA	10.58
Hoang TT et al., 2022, Japan (67)	38	NR	NR	3619 ± 2500	69	38.7	12/57	2386 ± 1935	SLE	5.75
Gerasimova EV et al., 2023, Russia (68)	100	47.67	12/88	5.73 ± 5.72	275	50.67	32/243	6.67 ± 7.53	RA	10.7
Chen JM et al., 2015, China (52)	205	NR	36/169	41.7 ± 13.2	220	NR	42/178	58.5 ± 22.8	SLE	NR
Mousa TG et al., 2018, Egypt (60)	50	NR	NR	0.8 ± 0.28	100	32.9	6/94	3.42 ± 1.4	SLE	NR
Román Fernández IV et al., 2019, Mexico (61)	10	NR	0/10	457.5 ± 83.45	38	48.2	0/38	510.2 ± 105.7	RA	8.35
Tapia-Llanos R et al., 2019, Mexico (63)	294	40	12/292	381 ± 202	293	37.4	18/275	394 ± 88	SLE	NR
Celik F et al., 2022, Turkey (66)	30	35.2	15/15	1.61 ± 0.32	60	36.1	35/25	8.05 ± 2.69	BD	NR

AS, ankylosing spondylitis; BD, Behcet’s disease; sCD40, soluble CD40; sCD40L, soluble CD40 ligand; CTD, connective tissue disease; MDD, mean disease duration; M/F, male-to-female ratio; NR, not reported; PsA, psoriatic arthritis; pSS, primary Sjogren syndrome; RA, rheumatoid arthritis; SLE, systemic lupus erythematosus; SpA, spondylarthritis; SSc, systemic sclerosis.

### sCD40L

#### Presence of RDs

Thirty-one studies, including 37 group comparators, investigated sCD40L concentrations in 2,414 RD patients (mean age 44.2 years, 87.8% females) and 1,384 healthy controls (mean age 42.3 years, 83.4% females) (34–51, 53–59, 61, 62, 64, 65, 67, 68). Thirteen studies were conducted in Europe (37, 38, 40, 43, 47, 49, 53, 57, 58, 62, 64, 65, 68), 11 in Asia (34, 36, 41, 42, 46, 48, 50, 51, 54, 55, 67), six in America (35, 39, 44, 56, 59, 61), and one in Africa (45). Systemic lupus erythematosus (SLE) patients were investigated in 15 study groups (34, 35, 38–40, 44, 45, 47, 51, 55, 57, 59, 64, 67), RA patients in eight (36, 38, 42, 47, 51, 61, 62, 68), BD patients in three (49, 53, 56), systemic sclerosis (SSc) patients in three (37, 41, 54), ankylosing spondylitis (AS) patients in three (48, 50, 58), primary Sjogren syndrome (pSS) patients in two (38, 47), psoriatic arthritis (PsA) patients in two (46, 65), and connective tissue disease (CTD) patients in one (43). sCD40L was measured in serum in 19 studies (35, 38–40, 42, 44–46, 48, 50, 51, 53, 54, 57, 58, 62, 65, 67, 68) and plasma in 11 (34, 36, 37, 41, 43, 47, 49, 55, 56, 61, 64). One study did not provide relevant information regarding the biological matrix used (59). An enzyme-linked immunosorbent assay (ELISA) was used in 25 studies (34–51, 53, 55, 56, 58, 61, 64, 68) and a platform for multi-analyte profiling in the remaining six (54, 57, 59, 62, 65, 67). Nineteen studies reported the mean RD duration, which ranged between 1.34 and 16.9 years (37, 39, 42, 43, 45, 47, 49, 51, 53, 55–57, 59, 61, 62, 64, 65, 67, 68). The risk of bias was low in 19 studies (36, 37, 39, 42, 43, 46–49, 51, 55–59, 62, 64, 65, 68), moderate in ten (34, 35, 38, 40, 45, 50, 53, 54, 61, 67), and high in two (41, 44) (**Supplementary Table 2**).

**Figure 2:**
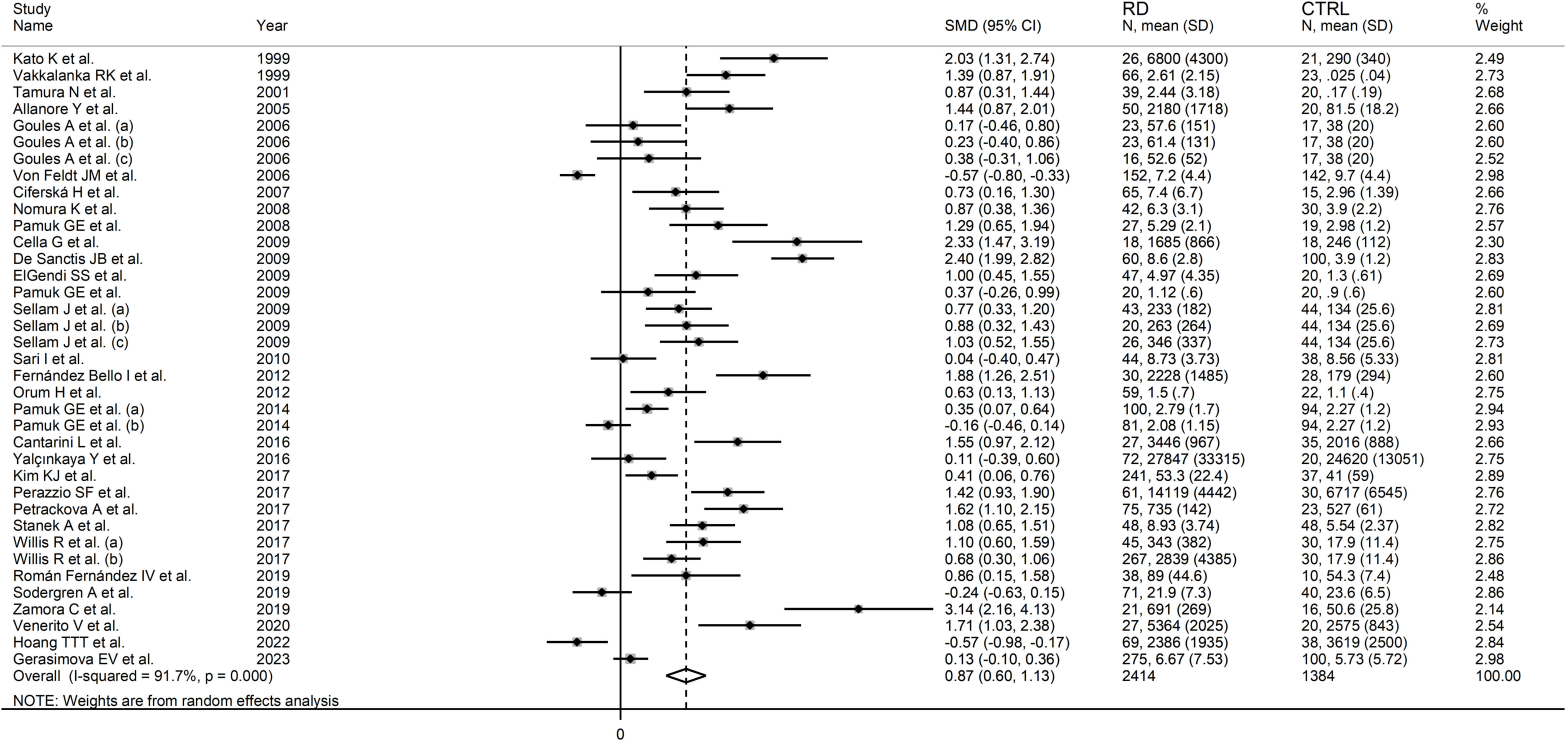
Forest plot of sCD40L concentrations in patients with rheumatic diseases and healthy controls.

**Figure 3:**
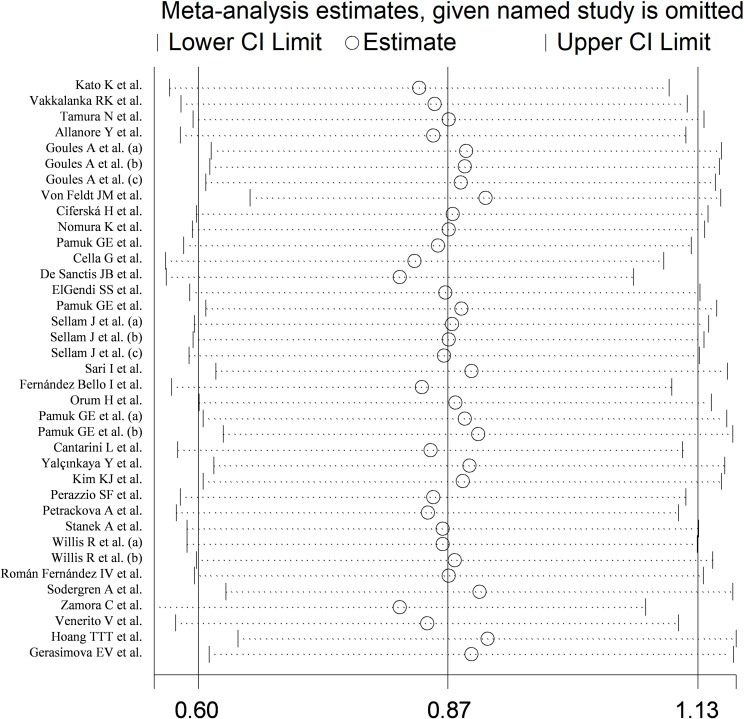
Sensitivity analysis of the association between sCD40L and rheumatic diseases.

The forest plot showed that sCD40L concentrations were significantly higher in RD patients than in controls (SMD=0.87, 95% CI 0.60 to 1.13, p<0.001; I^2^ = 91.7%, p<0.001; **Figure 2**). The meta-analysis results were stable in sensitivity analysis, with the corresponding pooled SMD values ranging between 0.82 and 0.91 (**Figure 3**).

**Figure 4:**
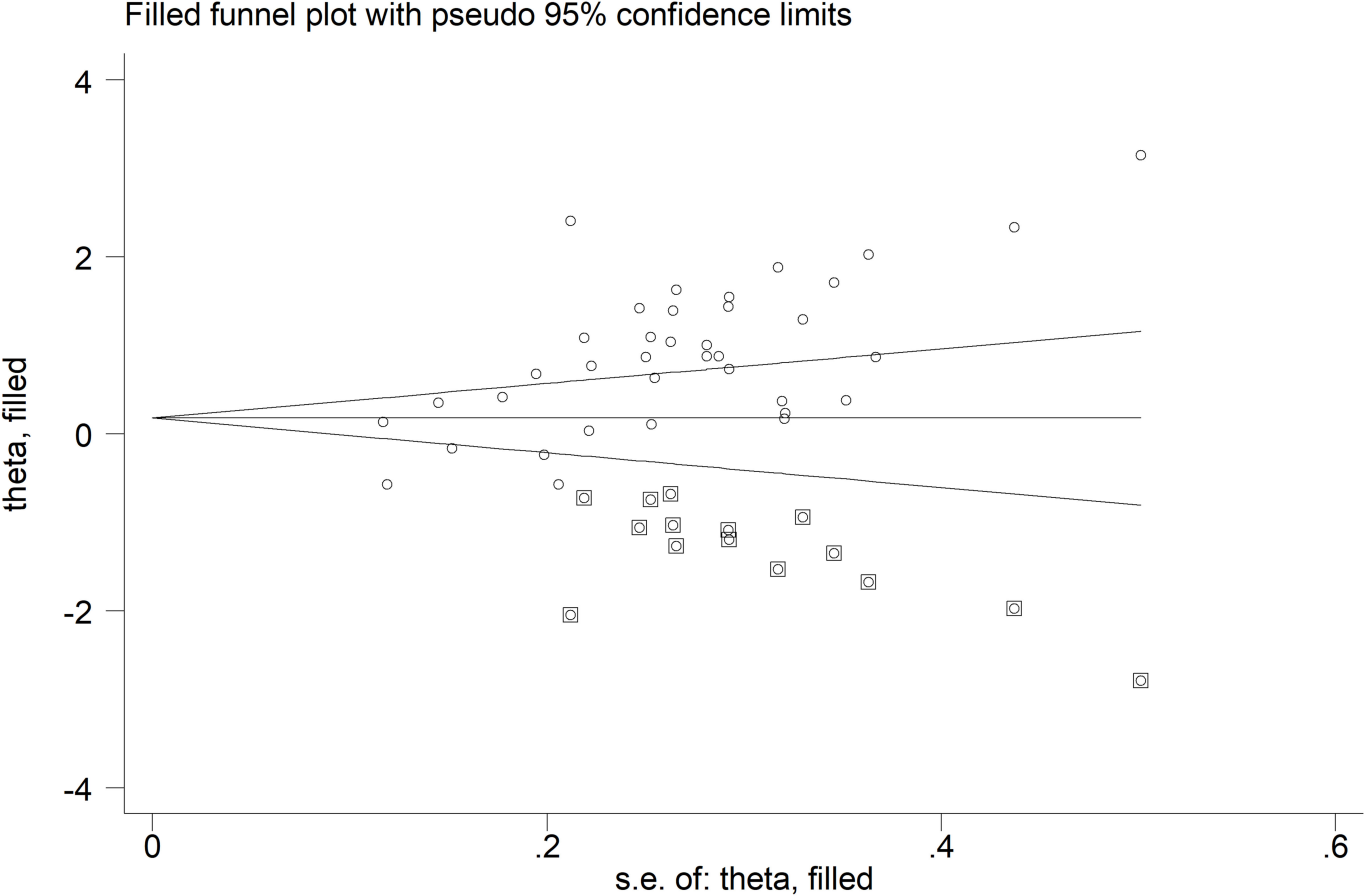
Funnel plot of the association between sCD40L and rheumatic diseases after “trimming-and-filling”. The enclosed circles and free circles represent dummy studies and genuine studies, respectively.

We observed a significant publication bias with Begg’s (p=0.002) and Egger’s (p<0.001) tests. The “trim-and-fill” method identified 15 missing studies to be added to the left side of the funnel plot to ensure symmetry (**Figure 4**). The resulting pooled SMD was significantly decreased and not significant (SMD=0.25, 95% CI -0.03 to 0.54, p=0.08).

**Figure 5:**
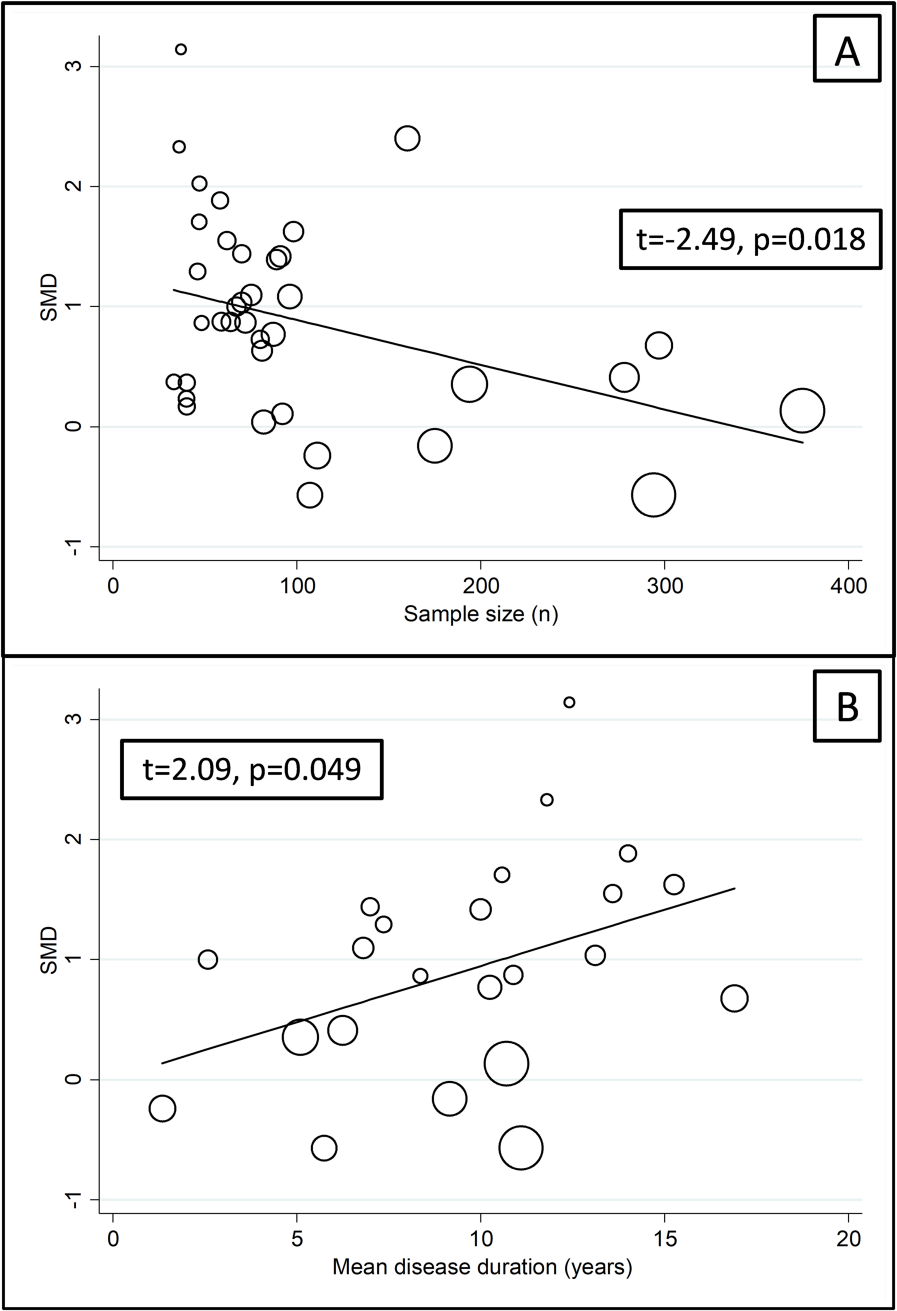
Bubble plot of the univariate meta-regression analysis between effect size and sample size **(A)** and mean disease duration **(B)**.

We did not observe significant associations in meta-regression analysis between the effect size and age (t=-0.93, p=0.36), male-to-female ratio (t=0.01, p=0.99), CRP (t=-0.23, p=0.82), ESR (t=-0.78, p=0.45), and use of DMARDs (t=-0.63, p=0.54) or glucocorticoids (t=0.18, p=0.86). By contrast, there was a significant negative association with sample size (t=-2.49, p=0.018; **Figure 5A**) and a positive association with the mean RD duration (t=2.09, p=0.049; **Figure 5B**).

**Figure 6:**
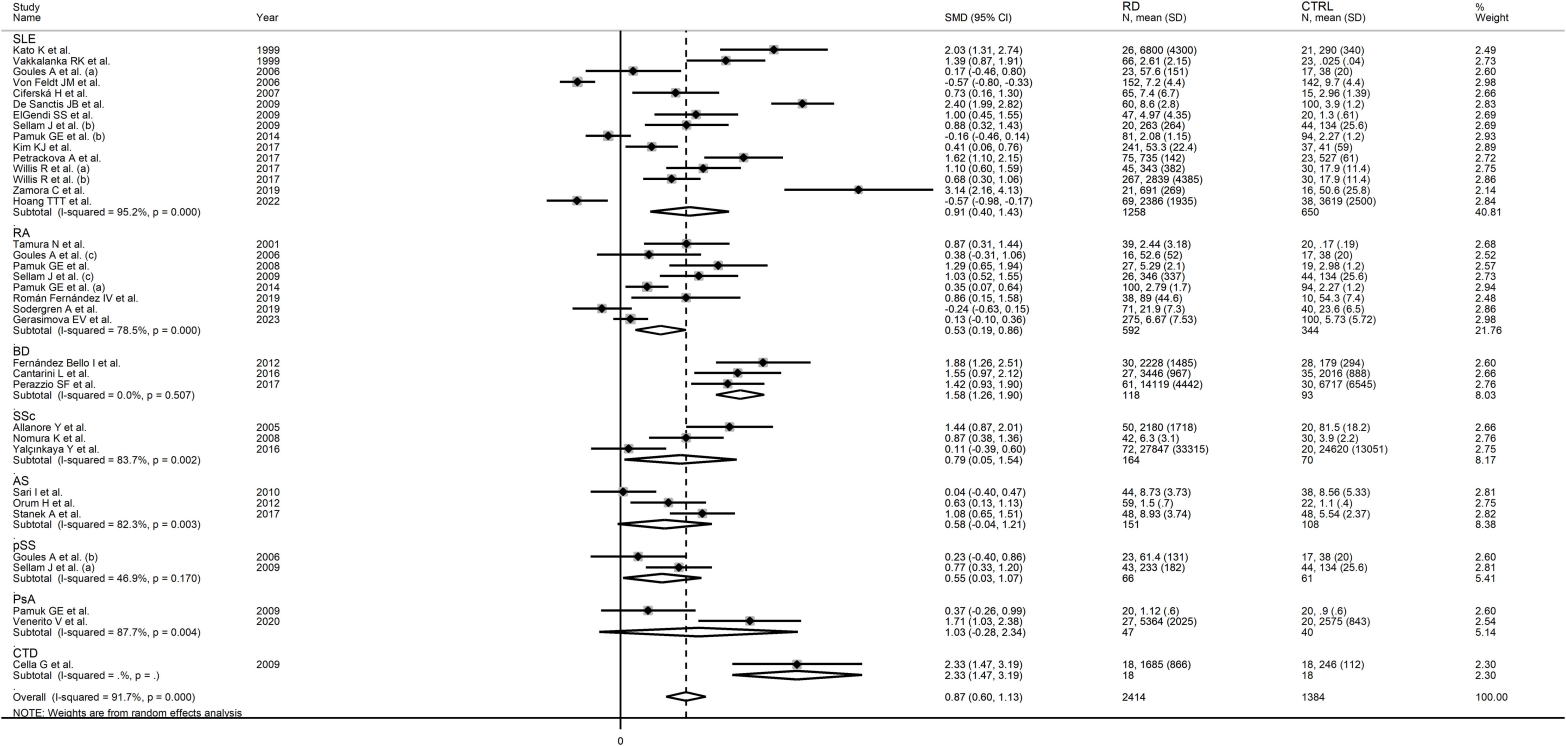
Forest plot of sCD40L concentrations in patients with rheumatic diseases and healthy controls according to the type of rheumatic disease.

**Figure 7:**
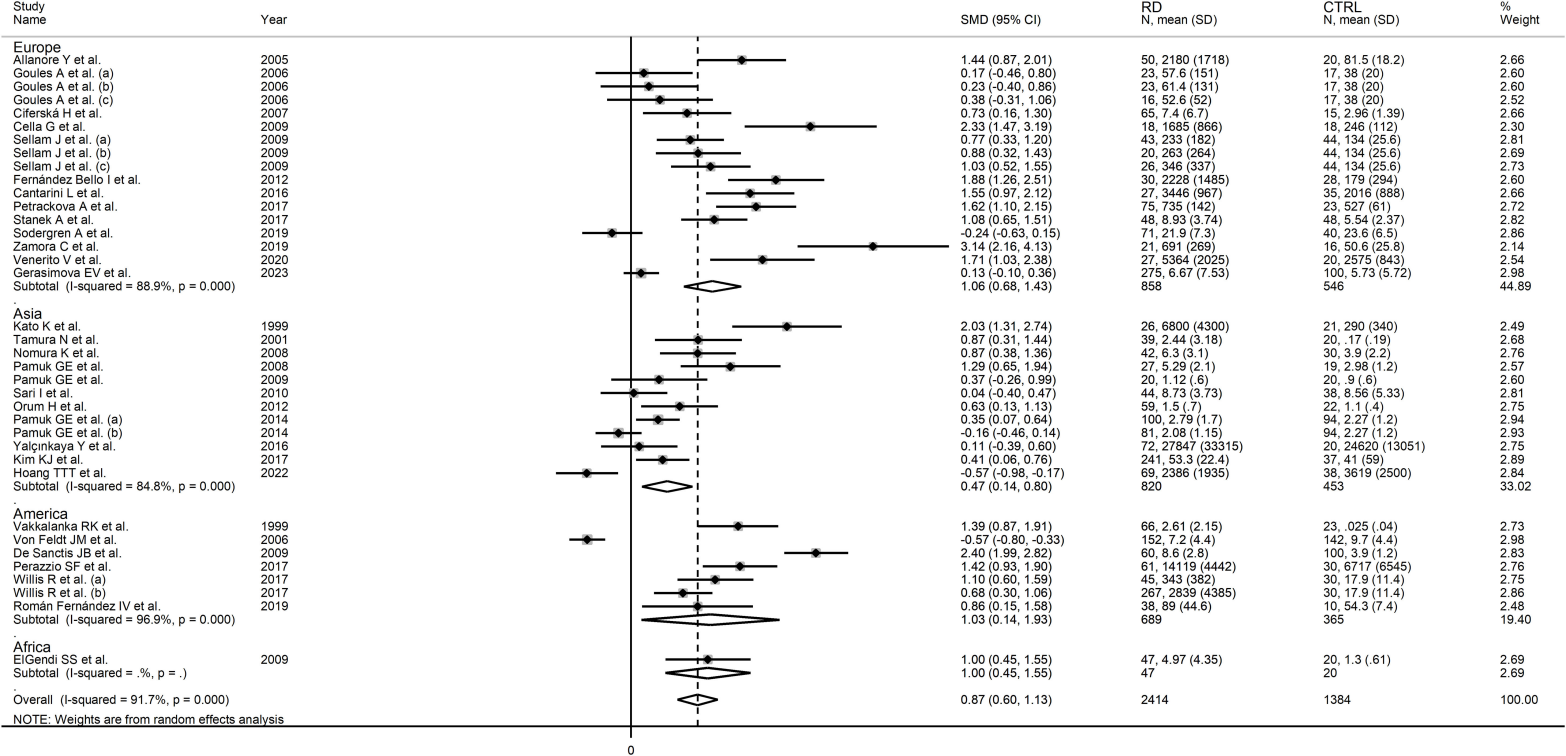
Forest plot of sCD40L concentrations in patients with rheumatic diseases and healthy controls according to the geographical area where the study was conducted.

**Figure 8:**
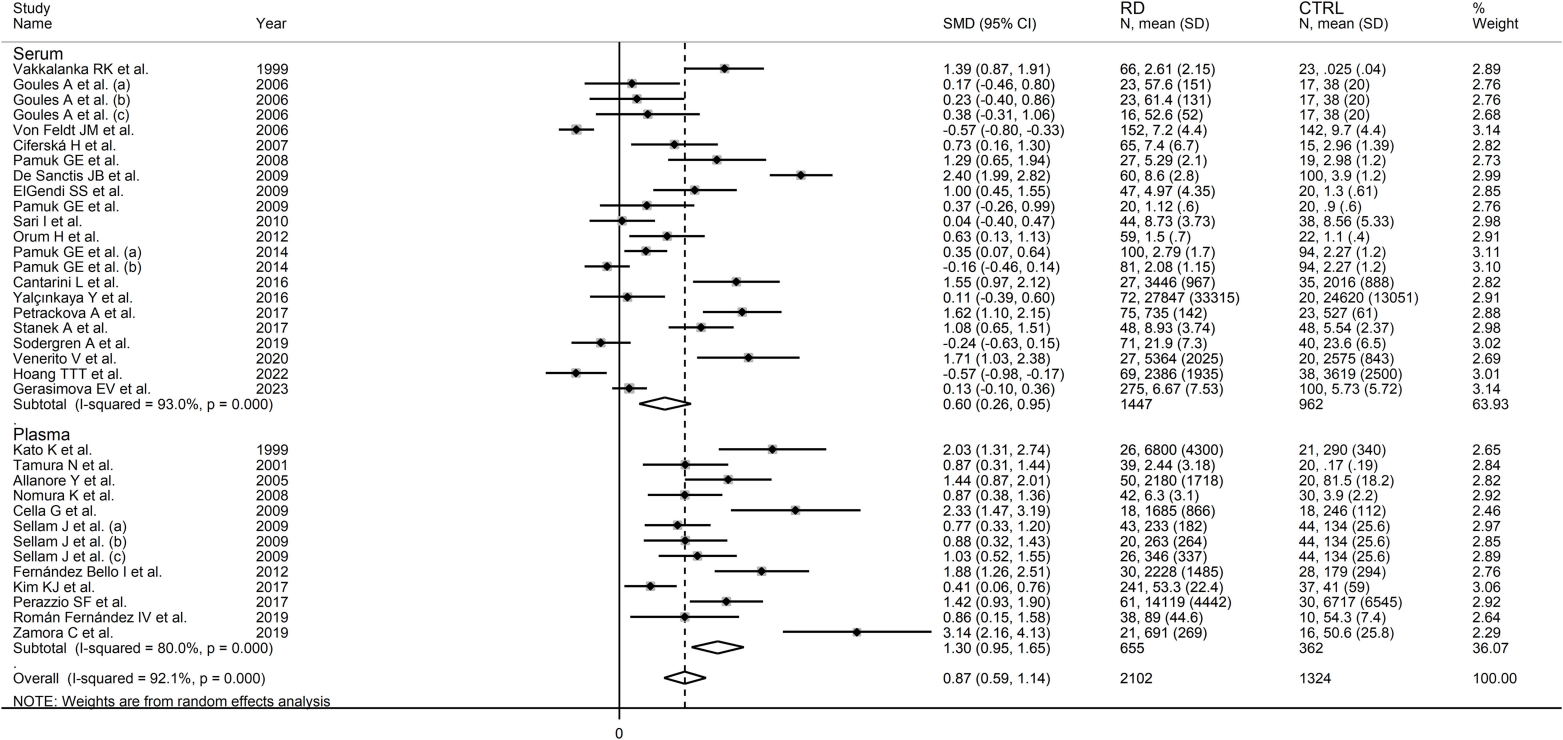
Forest plot of sCD40L concentrations in patients with rheumatic diseases and healthy controls according to the sample matrix assessed (serum or plasma).

**Figure 9:**
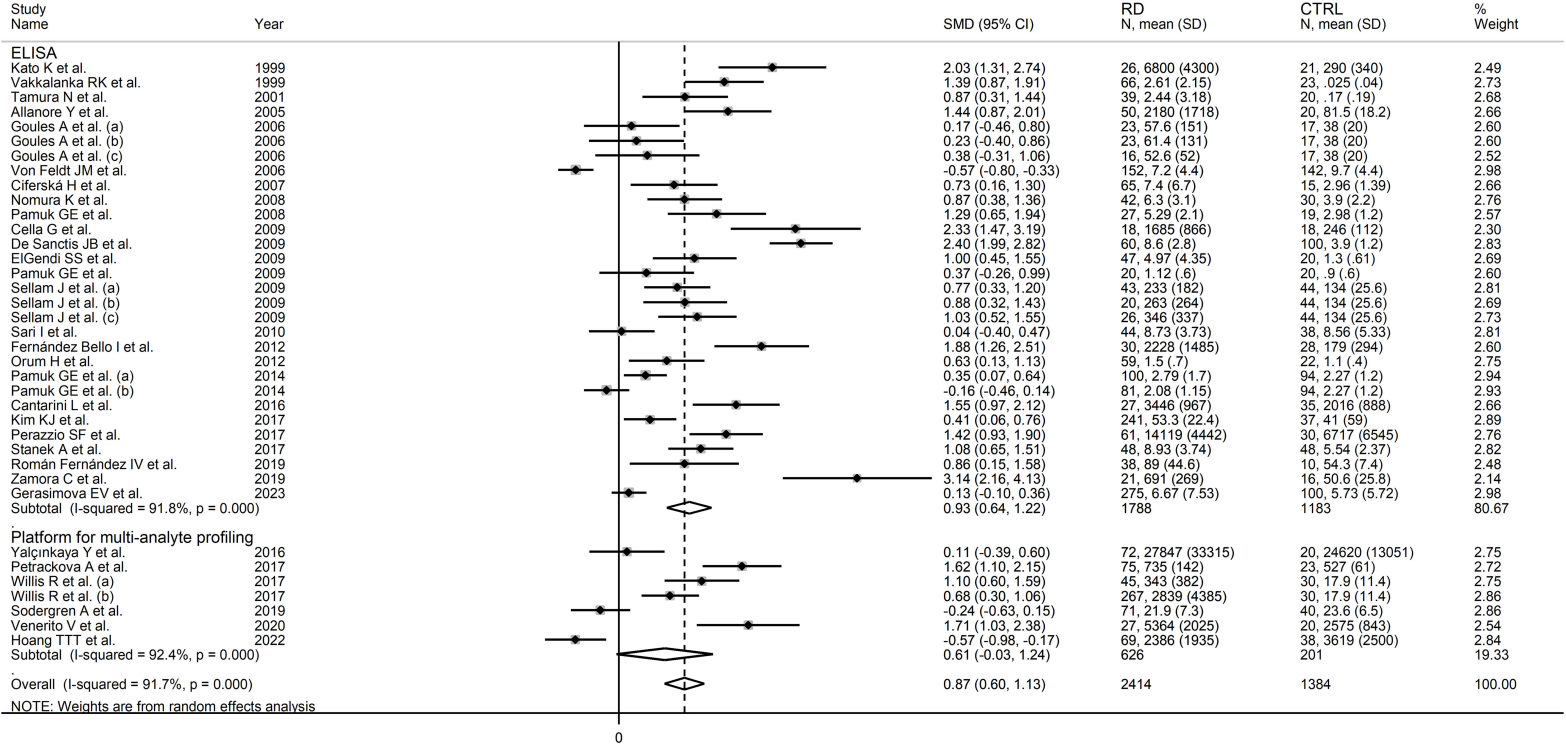
Forest plot of sCD40L concentrations in patients with rheumatic diseases and healthy controls according to the analytical method used.

In subgroup analysis, the pooled SMD was statistically significant in studies in SLE (SMD=0.91, 95% CI 0.40 to 1.43, p=0.001; I^2^ = 95.2%, p<0.001), RA (SMD=0.53, 95% CI 0.19 to 0.86, p=0.002; I^2^ = 78.5%, p<0.001), BD (SMD=1.58, 95% CI 1.26 to 1.90, p<0.001; I^2^ = 0.0%, p=0.51), SSc (SMD=0.79, 95% CI 0.05 to 1.54 p=0.036; I^2^ = 83.7%, p=0.002), and pSS patients (SMD=0.55, 95% CI 0.03 to 1.07 p=0.036; I^2^ = 46.9%, p=0.17), but not in AS (SMD=0.58, 95% CI -0.04 to 1.21, p=0.066; I^2^ = 82.3%, p=0.003) or PsA patients (SMD=1.03, 95% CI -0.28 to 2.34, p=0.12; I^2^ = 87.7%, p=0.004; **Figure 6**). In addition, the effect size in studies performed in BD patients was significantly larger than that in studies in RA (p=0.01), AS (p=0.047), and pSS patients (p=0.030), with a reduction of between-study variance in the BD (I^2^ = 0.0%) and pSS (I^2^ = 46.9%) subgroups. The pooled SMD was significant regardless of whether the studies were conducted in Europe (SMD=1.06, 95% CI 0.68 to 1.43, p<0.001; I^2^ = 88.9%, p<0.001), Asia (SMD=0.47, 95% CI 0.14 to 0.80, p=0.005; I^2^ = 84.8%, p<0.001), or America (SMD=1.03, 95% CI 0.14 to 1.93, p=0.024; I^2^ = 96.9%, p<0.001; **Figure 7**). There was a non-significant trend (p=0.06) toward a greater effect size in European studies compared to those conducted in Asia. The pooled SMD was significantly higher (p=0.013) in studies investigating plasma (SMD=1.30, 95% CI 0.95 to 1.65, p<0.001; I^2^ = 80.0%, p<0.001) compared to those in serum (SMD=0.60, 95% CI 0.26 to 0.95, p<0.001; I^2^ = 93.0%, p<0.001; **Figure 8**). Furthermore, the pooled SMD was significant in studies using ELISA (SMD=0.93, 95% CI 0.64 to 1.22, p<0.001; I^2^ = 91.8%, p<0.001) but not in those using a platform for multi-analyte profiling (SMD=0.61, 95% CI -0.03 to 1.24, p=0.061; I^2^ = 92.4%, p<0.001; **Figure 9**).

The overall level of the certainty of evidence remained low (level 2) after considering the low-moderate risk of bias in most studies (no change), the extreme but partially explainable heterogeneity (no change), the lack of indirectness (no change), the large effect size (SMD=0.87, upgrade one level) (69), and the presence of publication bias which was not addressed using the “trim-and-fill” method (downgrade one level).

#### Disease activity

Eight studies investigated sCD40L concentrations in 192 RD patients with active disease and 172 without (35, 40, 42, 45, 50, 53, 56, 61). Three focused on patients with SLE (35, 40, 45), two with RA (42, 61), two with BD (53, 56), and one with AS (50). The risk of bias was low in two studies (42, 56) and moderate in the remaining six (35, 40, 45, 50, 53, 61) (**Supplementary Table 2**).

**Figure 10:**
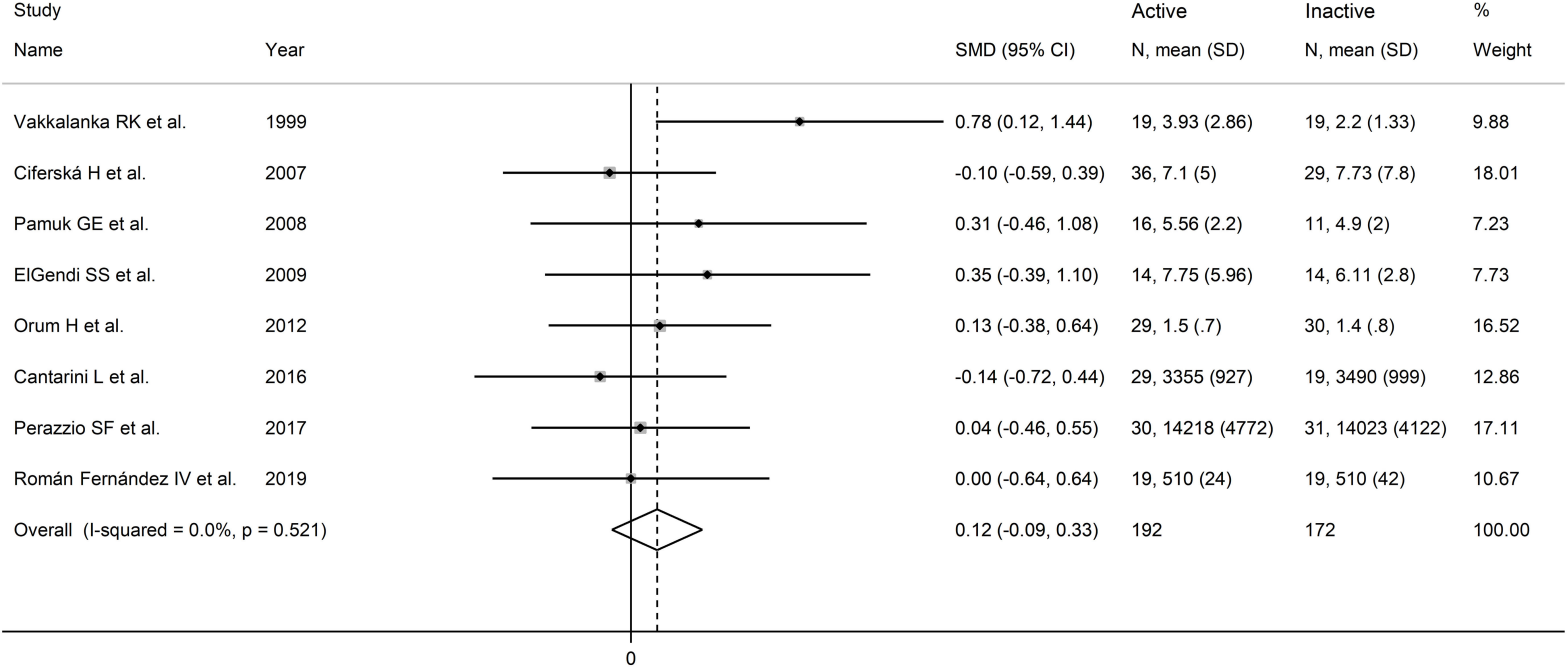
Forest plot of sCD40L concentrations in patients with rheumatic disease with and without active disease.

**Figure 11:**
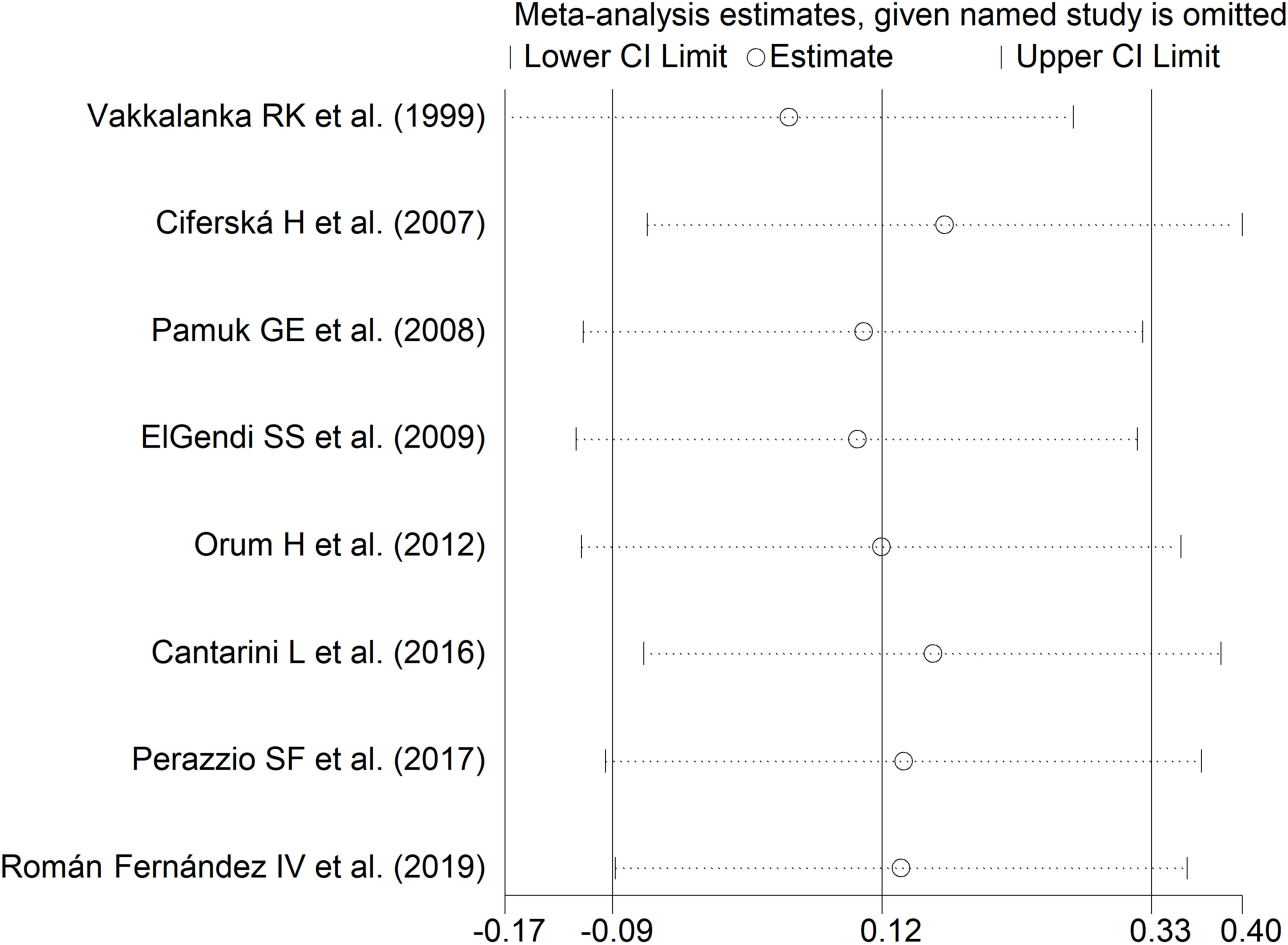
Sensitivity analysis of the association between sCD40L and active disease.

The forest plot showed no significant difference in sCD40L concentrations between RD patients with and without active disease (SMD=0.12, 95% CI -0.09 to 0.33, p=0.26; I^2^ = 0.0%, p=0.52; **Figure 10**). The results were stable in sensitivity analysis, with pooled SMD values ranging between 0.05 and 0.17 (**Figure 11**). The overall level of the certainty of evidence was downgraded to very low (level 1) as the relatively small number of studies prevented the assessment of publication bias and the conduct of meta-regression and subgroup analysis.

### sCD40

#### Presence of RDs

Five studies investigated sCD40 concentrations in 711 RD patients and 589 healthy controls (52, 60, 61, 63, 66). Two studies were conducted in Asia (52, 66), two in America (61, 63), and one in Africa (60). Three studies included patients with SLE (52, 60, 63), one with RA (61), and one with BD (66). An ELISA was used in all studies. Three studies measured serum (60, 63, 66) and the remaining two plasma (52, 61). The risk of bias was low in one study (66) and moderate in the remaining four (52, 60, 61, 63) (**Supplementary Table 2**).

**Figure 12:**
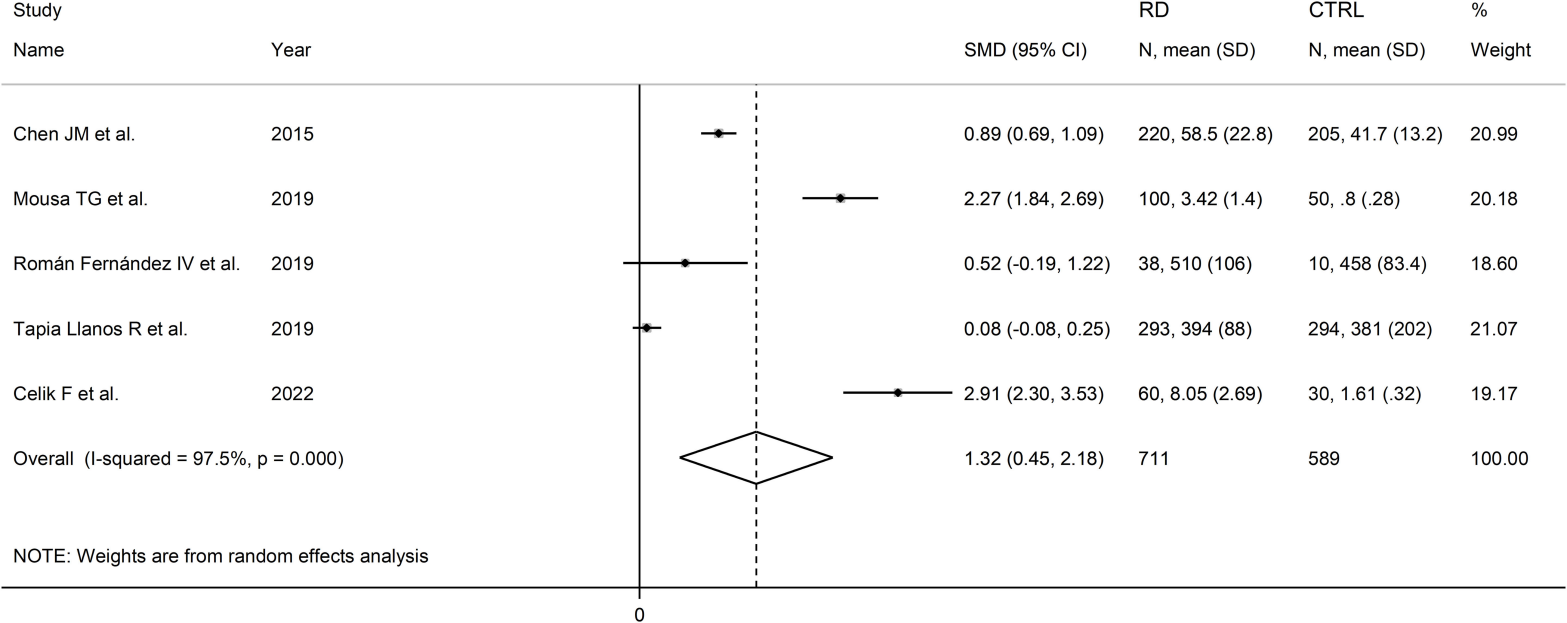
Forest plot of sCD40 concentrations in patients with rheumatic diseases and healthy controls.

The forest plot showed that sCD40 concentrations were significantly higher in RD patients than in controls (SMD=1.32, 95% CI 0.45 to 2.18, p=0.003; I^2^ = 97.5%, p<0.001; **Figure 12**).

**Figure 13:**
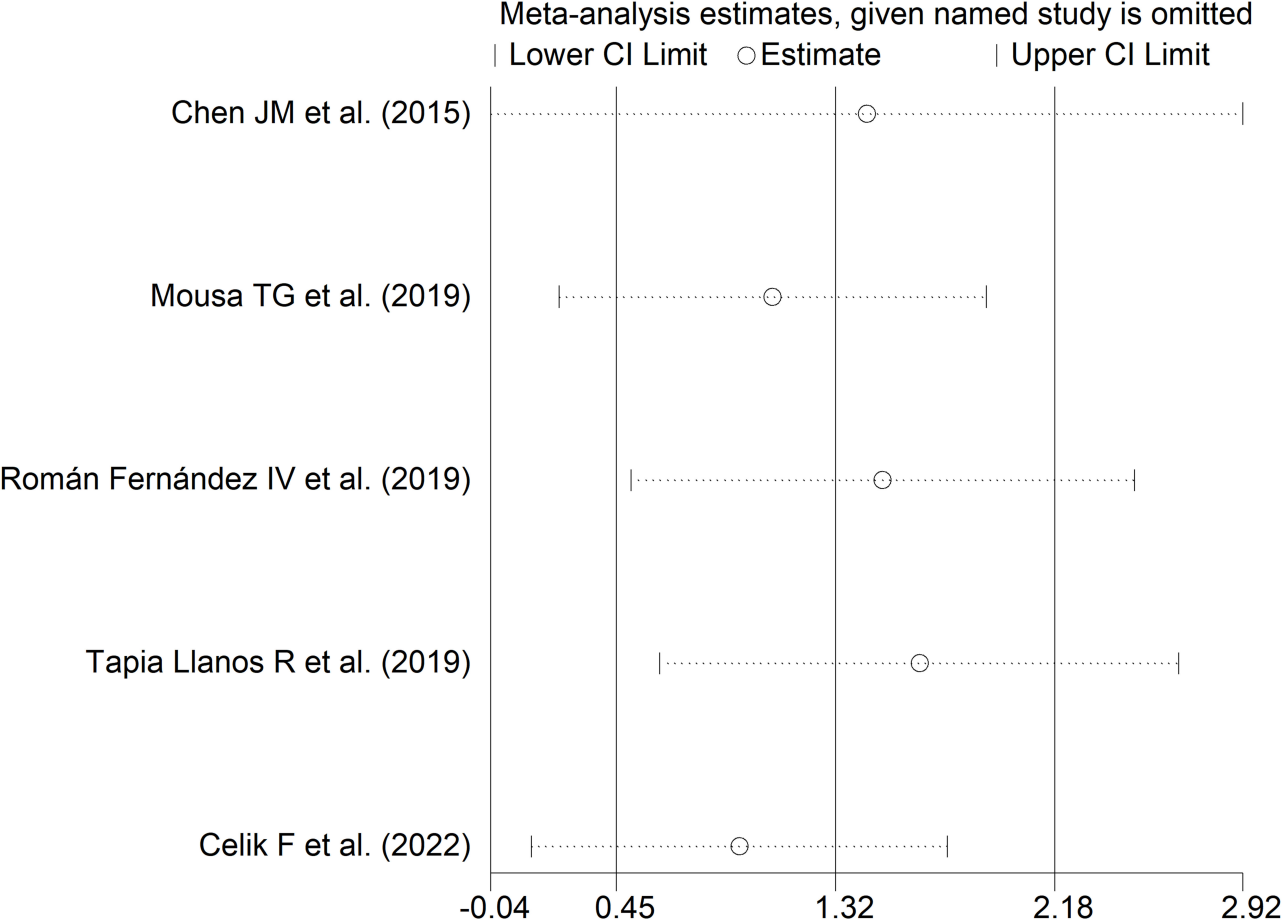
Sensitivity analysis of the association between sCD40 and rheumatic diseases.

Sensitivity analysis (SMD ranging between 0.94 and 0.65; **Figure 13**) showed that the effect size was not significant after excluding the study by Chen JM et al. (SMD=1.44; 95% CI -0.04 to 2.92; p=0.057; I^2^ = 98.0%, p<0.001) (52).

**Figure 14:**
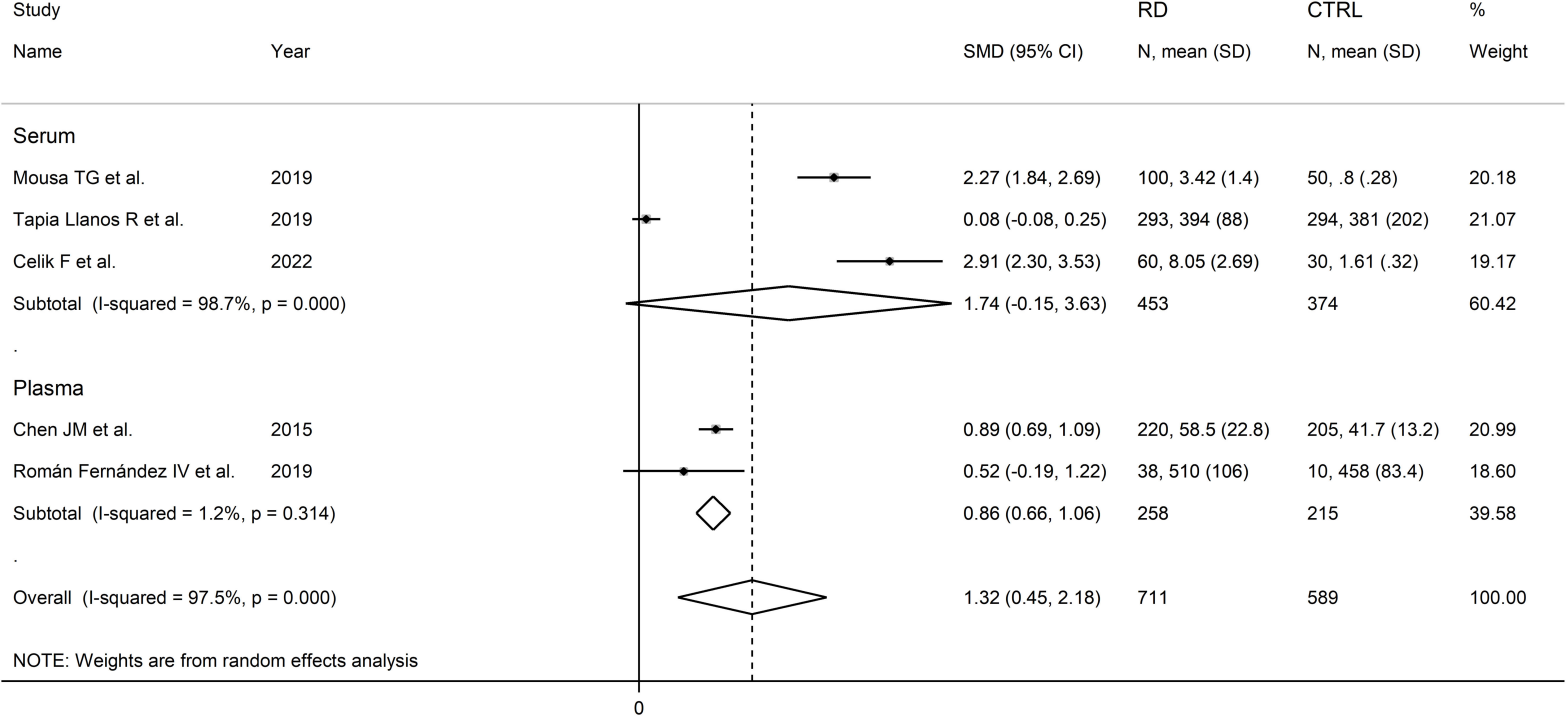
Forest plot of sCD40 concentrations in patients with rheumatic diseases and healthy controls according to the sample matrix assessed (serum or plasma).

In subgroup analysis, the pooled SMD was significant in studies measuring plasma (SMD=0.86, 95% CI 0.66 to 1.06, p<0.001; I^2^ = 1.2%, p=0.31) but not serum (SMD=1.74, 95% CI -0.15 to 3.63, p=0.072; I^2^ = 98.7%, p<0.001; **Figure 14**), with a virtually absent heterogeneity in the plasma subgroup.

Because of the small number of studies, assessment of publication bias and meta-regression could not be performed. Consequently, the overall level of the certainty of evidence was downgraded to very low (level 1).

#### Disease activity

Three studies investigated sCD40 concentrations in 135 RD patients with active disease and 82 without (61, 63, 66). Two studies were conducted in America (61, 63) and the remaining one in Asia (66). One study focused on RA patients (61), another on SLE patients (63), and the third on BD patients (66). An ELISA was used in all studies. The risk of bias was low in one study (66) and moderate in the other two (61, 63) (**Supplementary Table 2**).

**Figure 15:**
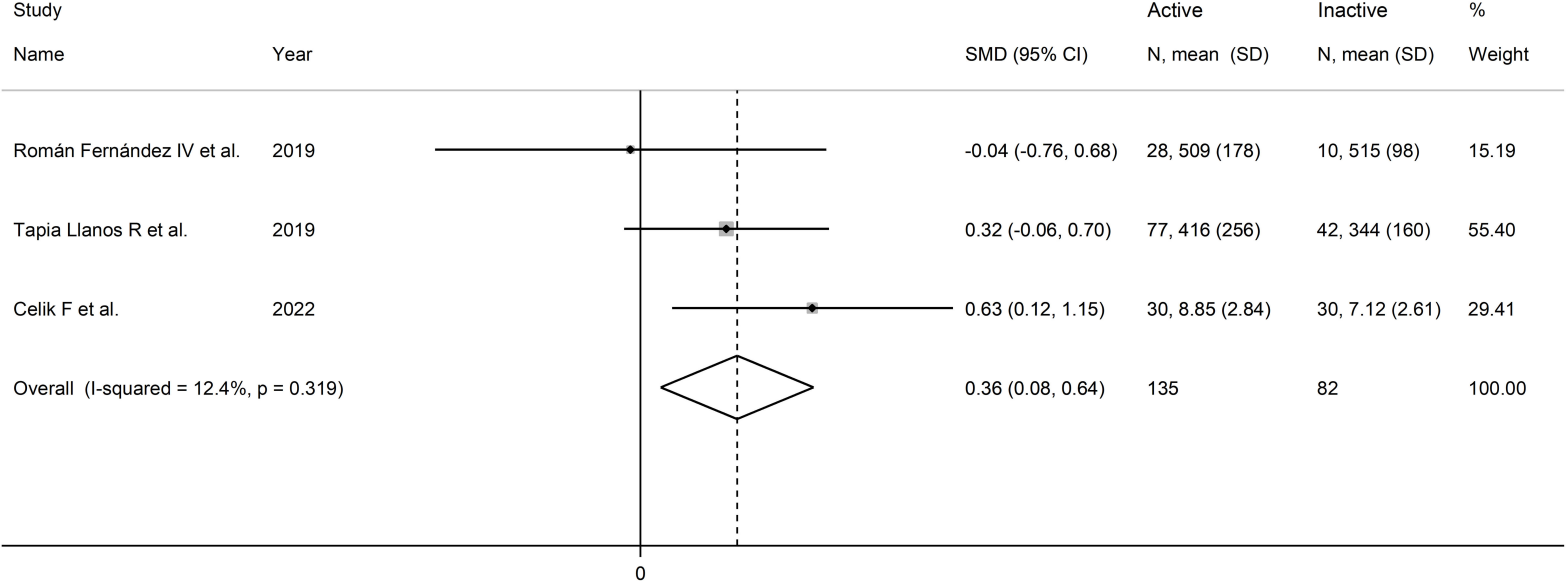
Forest plot of sCD40 concentrations in patients with rheumatic disease with and without active disease.

The forest plot showed that sCD40 concentrations were significantly higher in RD patients with active disease than in those with inactive disease (SMD=0.36, 95% CI 0.08 to 0.84, p=0.013; I^2^ = 12.4%, p=0.32; **Figure 15**).

The small number of studies prevented sensitivity analysis and the conduct of meta-regression and subgroup analysis, consequently downgrading the final level of the certainty of evidence to very low (level 1).

## Discussion

In this systematic review and meta-analysis, we observed significant RD-associated alterations in circulating sCD40 and sCD40L, critical humoral and cellular immune response regulators. Specifically, RD patients had significantly higher sCD40L concentrations when compared to healthy controls. However, the results require confirmation in further studies because of the observed publication bias and the absence of significant between-group differences after using the “trim-and-fill method”. In meta-regression, we did not observe significant associations between the effect size of the between-group differences in sCD40L concentrations and various demographic and clinical characteristics, particularly CRP, ESR, and use of DMARDs or glucocorticoids. However, there was a significant inverse association with the study sample size and a positive association with the mean RD duration. In subgroup analysis, the elevations in sCD40L concentrations were consistent across different types of RD (SLE, RA, BD, SSc, pSS, AS, and PsA), although they were not statistically significant in patients with AS and PsA. Furthermore, such elevations were observed in studies conducted in different geographical locations. Significant differences in the effect size were observed according to the biological matrix and the analytical method used. By contrast, we did not observe any between-group difference in circulating sCD40L between RD patients with and without active disease. In further analyses, RD patients had significant elevations in circulating sCD40 concentrations compared to controls, although the observed differences were primarily driven by one study in sensitivity analysis (52). Active disease was also associated with significant elevations in circulating sCD40. Albeit the limitations described warrant some caution, our study suggests that measuring sCD40L and sCD40 is worthy of further investigation to determine their role as candidate biomarkers of RDs.

One potential advantage of measuring sCD40L over conventional biomarkers of inflammation (e.g., CRP and ESR) is its capacity to reflect alterations in immune response in the context of autoimmune and autoinflammatory disorders (19, 20). The sCD40L-mediated CD40 intracellular signaling is initiated by members of the TNF receptor-associated factor (TRAF), which activates the canonical and non-canonical nuclear factor (NF)-κB pathway (70). This, in turn, leads to the nuclear translocation of p50/p65, p65/p65 and p52/RelB dimers and their DNA binding. Additional downstream pathways activated by the CD40-TRAF interaction include the mitogen-activated protein kinase, phosphoinositide-3-kinase-protein kinase B, and Janus kinase 3-signal transducer and activator of transcription pathways (71–73). The absence of significant associations in meta-regression between the effect size of sCD40L and CRP and ESR supports the proposition that measuring sCD40L may provide complementary information to conventional biomarkers of inflammation.

The observed elevations in circulating sCD40 in RD patients and in those with active disease are counterintuitive, given that sCD40 inhibits the interaction between CD40L and mCD40 and can be considered a negative control feedback mechanism to prevent excess activation of mCD40 (19, 20). However, an additional element of complexity is related to the role of a disintegrin and metalloprotease 17 (ADAM17), involved in various functions, including CD40 ectodomain shedding and the release of sCD40 in B cells and endothelial cells (74, 75). Notably, some studies have reported an anti-inflammatory effect of ADAM17 by shedding adhesion molecules and the TNF receptor (76–78), whereas other studies suggest a proinflammatory effect (79, 80). Further research is therefore required to investigate whether sCD40 can exert opposing effects on immune and inflammatory pathways in patients with RDs, including those with active disease.

While our analyses suggest a potential role of sCD40 and sCD40L as biomarkers of different types of RDs, further studies are required to confirm these findings and justify their utility in routine clinical practice. Larger, accurately designed prospective studies should investigate the diagnostic performance in a wider range of autoimmune, mixed autoimmune-autoinflammatory, and autoinflammatory RDs (1–4). Such performance should be compared to existing diagnostic criteria, serological biomarkers, and non-specific markers of inflammation in individual RDs to determine whether measuring circulating sCD40 and sCD40L significantly enhances diagnosis over and above available tools.

Our systematic review and meta-analysis has several strengths, including the comprehensive assessment of sCD40 and sCD40L in different RDs, the evaluation of the level of the certainty of evidence for each studied endpoint (presence of RD and active disease), and the study of possible associations between the effect size and various study and patient characteristics. Significant limitations are the relatively low number of studies investigating sCD40 and the cross-sectional design of the selected studies, which did not allow for the investigation of a cause-effect relationship between sCD40 and sCD40L and RDs and active disease.

In conclusion, our study has shown that patients with RDs have significantly elevated circulating concentrations of sCD40 and sCD40L when compared to healthy controls. Such alterations likely reflect a dysregulated humoral and cellular immune response and are not associated with elevations in conventional inflammatory biomarkers, i.e., CRP and ERS. Further prospective studies in a broader range of RDs are required to establish whether measuring sCD40 and sCD40L can be helpful in the clinical evaluation and monitoring of RDs.

## Data Availability

The raw data supporting the conclusions of this article will be made available by the authors, without undue reservation.
