# Supplementary material for: sCD40 and sCD40L as candidate biomarkers of rheumatic diseases: a systematic review and meta-analysis with meta-regression
Source: Front Immunol. 2025 Mar 19;16:1479904. doi: 10.3389/fimmu.2025.1479904 (PMC11962221; doi:10.3389/fimmu.2025.1479904)
Supplement: Supplementary file 2 [file Table2.docx]

**Supplementary Table 2.** Assessment of the risk of bias using the Joanna Briggs Institute critical appraisal checklist.

| **Study** | **Were the inclusion criteria clearly defined?** | **Were the subjects and the setting described in detail?** | **Was the exposure measured in a reliable way?** | **Were standard criteria used to assess the condition?** | **Were confounding factors identified?** | **Were strategies to deal with confounding factors stated?** | **Were the outcomes measured in a reliable way?** | **Was appropriate statistical analysis used?** | **Risk of bias** |
| --- | --- | --- | --- | --- | --- | --- | --- | --- | --- |
| Kato K et al. (1) | No | No | Yes | Yes | No | No | Yes | Yes | Moderate |
| Vakkalanka RK et al. (2) | No | Yes | Yes | Yes | No | No | Yes | Yes | Moderate |
| Tamura N et al. (3) | Yes | Yes | Yes | Yes | No | No | Yes | Yes | Low |
| Allanore Y et al. (4) | Yes | Yes | Yes | Yes | No | No | Yes | Yes | Low |
| Goules A et al. (5) | No | No | Yes | Yes | No | No | Yes | Yes | Moderate |
| Von Feldt JM et al. (6) | No | No | Yes | Yes | Yes | Yes | Yes | Yes | Low |
| Ciferská H et al. (7) | No | No | Yes | Yes | No | No | Yes | Yes | Moderate |
| Nomura K et al. (8) | No | No | Yes | No | No | No | Yes | Yes | High |
| Pamuk GE et al. (9) | Yes | Yes | Yes | Yes | No | No | Yes | Yes | Low |
| Cella G et al. (10) | Yes | Yes | Yes | Yes | No | No | Yes | Yes | Low |
| De Sanctis JB et al. (11) | No | No | Yes | No | No | No | Yes | Yes | High |
| ElGendi SS et al. (12) | No | Yes | Yes | Yes | No | No | Yes | Yes | Moderate |
| Pamuk GE et al. (13) | Yes | Yes | Yes | Yes | No | No | Yes | Yes | Low |
| Sellam J et al. (14) | Yes | Yes | Yes | Yes | No | No | Yes | Yes | Low |
| Sari I et al. (15) | Yes | Yes | Yes | Yes | No | No | Yes | Yes | Low |
| Fernández Bello I et al. (16) | Yes | Yes | Yes | Yes | No | No | Yes | Yes | Low |
| Orum H et al. (17) | No | Yes | Yes | Yes | No | No | Yes | Yes | Moderate |
| Pamuk GE et al. (18) | No | Yes | Yes | Yes | Yes | Yes | Yes | Yes | Low |
| Cantarini L et al. (19) | No | Yes | Yes | Yes | No | No | Yes | Yes | Moderate |
| Yalçınkaya Y et al. (20) | No | Yes | Yes | Yes | No | No | Yes | Yes | Moderate |
| Kim KJ et al. (21) | Yes | Yes | Yes | Yes | Yes | Yes | Yes | Yes | Low |
| Perazzio SF et al. (22) | Yes | Yes | Yes | Yes | No | No | Yes | Yes | Low |
| Petrackova A et al. (23) | Yes | Yes | Yes | Yes | No | No | Yes | Yes | Low |
| Stanek A et al. (24) | Yes | Yes | Yes | Yes | No | No | Yes | Yes | Low |
| Willis R et al. (25) | Yes | Yes | Yes | No | Yes | Yes | Yes | Yes | Low |
| Román Fernández IV et al. (26) | No | Yes | Yes | Yes | No | No | Yes | Yes | Moderate |
| Sodergren A et al. (27) | No | Yes | Yes | Yes | Yes | Yes | Yes | Yes | Low |
| Zamora C et al. (28) | Yes | Yes | Yes | Yes | No | No | Yes | Yes | Low |
| Venerito V et al. (29) | No | No | Yes | Yes | Yes | Yes | Yes | Yes | Low |
| Hoang TT et al. (30) | No | Yes | Yes | Yes | No | No | Yes | Yes | Moderate |
| Gerasimova EV et al. (31) | Yes | Yes | Yes | Yes | No | No | Yes | Yes | Low |
| Chen JM et al. (32) | No | Yes | Yes | No | No | No | Yes | Yes | Moderate |
| Mousa TG et al. (33) | No | Yes | Yes | Yes | No | No | Yes | Yes | Moderate |
| Tapia-Llanos R et al. (34) | No | Yes | Yes | Yes | No | No | Yes | Yes | Moderate |
| Celik F et al. (35) | Yes | Yes | Yes | Yes | No | No | Yes | Yes | Low |

**References**

1. Kato K, Santana-Sahagun E, Rassenti LZ, Weisman MH, Tamura N, Kobayashi S, et al. The soluble CD40 ligand sCD154 in systemic lupus erythematosus. J Clin Invest. 1999;104(7):947-55. doi: 10.1172/JCI7014

2. Vakkalanka RK, Woo C, Kirou KA, Koshy M, Berger D, Crow MK. Elevated levels and functional capacity of soluble CD40 ligand in systemic lupus erythematosus sera. Arthritis Rheum. 1999;42(5):871-81. doi: 10.1002/1529-0131(199905)42:5<871::AID-ANR5>3.0.CO;2-J

3. Tamura N, Kobayashi S, Kato K, Bando H, Haruta K, Oyanagi M, et al. Soluble CD154 in rheumatoid arthritis: elevated plasma levels in cases with vasculitis. J Rheumatol. 2001;28(12):2583-90. doi:

4. Allanore Y, Borderie D, Meune C, Lemaréchal H, Ekindjian O, Kahan A. Increased plasma soluble CD40 ligand concentrations in systemic sclerosis and association with pulmonary arterial hypertension and digital ulcers. Ann Rheum Dis. 2005;63(3):109-. doi: 10.1136/ard.2003.020040

5. Goules A, Tzioufas AG, Manousakis MN, Kirou KA, Crow MK, Routsias JG. Elevated levels of soluble CD40 ligand (sCD40L) in serum of patients with systemic autoimmune diseases. J Autoimmun. 2006;26(3):165-71. doi: 10.1016/j.jaut.2006.02.002

6. Von Feldt JM, Scalzi LV, Cucchiara AJ, Morthala S, Kealey C, Flagg SD, et al. Homocysteine levels and disease duration independently correlate with coronary artery calcification in patients with systemic lupus erythematosus. Arthritis Rheum. 2006;54(7):2220-7. doi: 10.1002/art.21967

7. Ciferská H, Horák P, Heřmanová Z, Ordeltová M, Zadražil J, Tichý T, et al. The levels of sCD30 and of sCD40L in a group of patients with systemic lupus erythematodes and their diagnostic value. Clin Rheumatol. 2007;26(5):723-8. doi: 10.1007/s10067-006-0389-9

8. Nomura S, Inami N, Ozaki Y, Kagawa H, Fukuhara S. Significance of microparticles in progressive systemic sclerosis with interstitial pneumonia. Platelets. 2008;19(3):192-8. doi: 10.1080/09537100701882038

9. Pamuk GE, Vural Ö, Turgut B, Demır M, Pamuk ÖN, Çakir N. Increased platelet activation markers in rheumatoid arthritis: Are they related with subclinical atherosclerosis? Platelets. 2008;19(2):146-54. doi: 10.1080/09537100701210057

10. Cella G, Vianello F, Cozzi F, Marotta H, Tona F, Saggiorato G, et al. Effect of bosentan on plasma markers of endothelial cell activity in patients with secondary pulmonary hypertension related to connective tissue diseases. J Rheumatol. 2009;36(4):760-7. doi: 10.3899/jrheum.080542

11. de Sanctis JB, Garmendia JV, Chaurio R, Zabaleta M, Rivas L. Total and biologically active CD154 in patients with SLE. Autoimmunity. 2009;42(4):263-5. doi: 10.1080/08916930902827942

12. ElGendi SS, El-Sherif WT. Anti-C1q antibodies, sCD40L, TWEAK and CD4/CD8 ratio in systemic lupus erythematosus and their relations to disease activity and renal involvement. Egypt J Immunol. 2009;16(1):135-48. doi:

13. Pamuk GE, Nuri Pamuk O, Orum H, Arican O, Turgut B, Demir M. Elevated platelet-monocyte complexes in patients with psoriatic arthritis. Platelets. 2009;20(7):493-7. doi: 10.3109/09537100903165174

14. Sellam J, Proulle V, Jüngel A, Ittah M, Richard CM, Gottenberg JE, et al. Increased levels of circulating microparticles in primary Sjogren's syndrome, systemic lupus erythematosus and rheumatoid arthritis and relation with disease activity. Arthritis Res Ther. 2009;11(5). doi: 10.1186/ar2833

15. Sari I, Alacacioglu A, Kebapcilar L, Taylan A, Bilgir O, Yildiz Y, et al. Assessment of soluble cell adhesion molecules and soluble CD40 ligand levels in ankylosing spondylitis. Joint Bone Spine. 2010;77(1):85-7. doi: 10.1016/j.jbspin.2009.07.005

16. Fernández Bello I, Álvarez MT, López-Longo FJ, Arias-Salgado EG, Martín M, Jiménez-Yuste V, et al. Platelet soluble CD40L and matrix metalloproteinase 9 activity are proinflammatory mediators in Behçet disease patients. Thromb Haemost. 2012;107(01):88-98. doi: 10.1160/th11-08-0556

17. Örüm H, Pamuk GE, Pamuk ÖN, Demir M, Turgut B. Does anti-tnf therapy cause any change in platelet activation in ankylosing spondylitis patients? J Thromb Thrombolysis. 2012;33(2):154-9. doi: 10.1007/s11239-011-0663-9

18. Pamuk ON, Tozkir H, Uyanik MS, Gurkan H, Saritas F, Duymaz J, et al. PECAM-1 gene polymorphisms and soluble PECAM-1 level in rheumatoid arthritis and systemic lupus erythematosus patients: any link with clinical atherosclerotic events? Clin Rheumatol. 2014;33(12):1737-43. doi: 10.1007/s10067-014-2771-3

19. Cantarini L, Pucino V, Vitale A, Talarico R, Lucherini OM, Magnotti F, et al. Immunometabolic biomarkers of inflammation in Behcet's disease: relationship with epidemiological profile, disease activity and therapeutic regimens. Clin Exp Immunol. 2016;184(2):197-207. doi: 10.1111/cei.12768

20. Yalcinkaya Y, Cinar S, Artim-Esen B, Kamali S, Ocal L, Deniz G, et al. The relationship between vascular biomarkers and disease characteristics in systemic sclerosis: elevated MCP-1 is predominantly associated with fibrotic manifestations. Clin Exp Rheumatol. 2016;34 Suppl 100(5):110-4. doi:

21. Kim KJ, Baek IW, Yoon CH, Kim WU, Cho CS. Elevated levels of soluble CD40 ligand are associated with antiphospholipid antibodies in patients with systemic lupus erythematosus. Clin Exp Rheumatol. 2017;35(5):823-30. doi:

22. Perazzio SF, Soeiro-Pereira PV, dos Santos VC, de Brito MV, Salu B, Oliva MLV, et al. Soluble CD40L is associated with increased oxidative burst and neutrophil extracellular trap release in Behcet's disease. Arthritis Res Ther. 2017;19(1). doi: 10.1186/s13075-017-1443-5

23. Petrackova A, Smrzova A, Gajdos P, Schubertova M, Schneiderova P, Kromer P, et al. Serum protein pattern associated with organ damage and lupus nephritis in systemic lupus erythematosus revealed by PEA immunoassay. Clin Proteomics. 2017;14(1):32. doi: 10.1186/s12014-017-9167-8

24. Stanek A, Cholewka A, Wielkoszynski T, Romuk E, Sieron K, Sieron A. Increased Levels of Oxidative Stress Markers, Soluble CD40 Ligand, and Carotid Intima-Media Thickness Reflect Acceleration of Atherosclerosis in Male Patients with Ankylosing Spondylitis in Active Phase and without the Classical Cardiovascular Risk Factors. Oxid Med Cell Longev. 2017;2017:1-8. doi: 10.1155/2017/9712536

25. Willis R, Smikle M, DeCeulaer K, Romay-Penabad Z, Papalardo E, Jajoria P, et al. Clinical associations of proinflammatory cytokines, oxidative biomarkers and vitamin D levels in systemic lupus erythematosus. Lupus. 2017;26(14):1517-27. doi: 10.1177/0961203317706557

26. Román-Fernández IV, García-Chagollán M, Cerpa-Cruz S, Jave-Suárez LF, Palafox-Sánchez CA, García-Arellano S, et al. Assessment of CD40 and CD40L expression in rheumatoid arthritis patients, association with clinical features and DAS28. Clin Exp Med. 2019;19(4):427-37. doi: 10.1007/s10238-019-00568-5

27. Södergren A, Karp K, Bengtsson C, Möller B, Rantapää-Dahlqvist S, Wållberg-Jonsson S. Biomarkers associated with cardiovascular disease in patients with early rheumatoid arthritis. PLos One. 2019;14(8). doi: 10.1371/journal.pone.0220531

28. Zamora C, Toniolo E, Diaz-Torne C, Canto E, Magallares B, Ortiz MA, et al. Association of Platelet Binding to Lymphocytes with B Cell Abnormalities and Clinical Manifestations in Systemic Lupus Erythematosus. Mediators Inflamm. 2019;2019:2473164. doi: 10.1155/2019/2473164

29. Venerito V, Natuzzi D, Bizzoca R, Lacarpia N, Cacciapaglia F, Lopalco G, et al. Serum sCD40L levels are increased in patients with psoriatic arthritis and are associated with clinical response to apremilast. Clin Exp Immunol. 2020;201(2):200-4. doi: 10.1111/cei.13451

30. Hoang TTT, Ichinose K, Morimoto S, Furukawa K, Le LHT, Kawakami A. Measurement of anti-suprabasin antibodies, multiple cytokines and chemokines as potential predictive biomarkers for neuropsychiatric systemic lupus erythematosus. Clin Immunol. 2022;237. doi: 10.1016/j.clim.2022.108980

31. Gerasimova EV, Popkova TV, Gerasimova DA, Markina YV, Kirichenko TV. Subclinical Carotid Atherosclerosis in Patients with Rheumatoid Arthritis at Low Cardiovascular Risk. Biomedicines. 2023;11(3). doi: 10.3390/biomedicines11030974

32. Chen JM, Guo J, Wei CD, Wang CF, Luo HC, Wei YS, et al. The association of CD40 polymorphisms with CD40 serum levels and risk of systemic lupus erythematosus. BMC Genet. 2015;16(1):121. doi: 10.1186/s12863-015-0279-8

33. Mousa TG, Omar HH, Emad R, Salama MI, Omar W, Fawzy M, et al. The association of CD40 polymorphism (rs1883832C/T) and soluble CD40 with the risk of systemic lupus erythematosus among Egyptian patients. Clin Rheumatol. 2018;38(3):777-84. doi: 10.1007/s10067-018-4349-y

34. Tapia‐Llanos R, Muñoz‐Valle JF, Román‐Fernández IV, Marín‐Rosales M, Salazar‐Camarena DC, Cruz A, et al. Association of soluble CD40 levels with ‐1 C > T CD40 polymorphism and chronic kidney disease in systemic lupus erythematosus. Mol Genet Genomic Med. 2019;7(12). doi: 10.1002/mgg3.1014

35. Celik F, Coteli E, Gul FC, Ozsoy E, Kobat SG, Karagoz ZK, et al. Interleukin 18, soluble cluster of differentiation 40, platelet factor 4 variant 1, and neutrophil gelatinase-associated lipocalin can be used as biomarkers to aid activity and diagnosis in ocular Behcet's disease. Int Ophthalmol. 2022;42(11):3321-31. doi: 10.1007/s10792-022-02331-4
